# Supplementary figures and images for: PixlMap: A generalisable pixel classifier for cellular phenotyping in multiplex immunofluorescence images
Source: PLoS One. 2025 Dec 3;20(12):e0317865. doi: 10.1371/journal.pone.0317865 (PMC12674528; doi:10.1371/journal.pone.0317865)

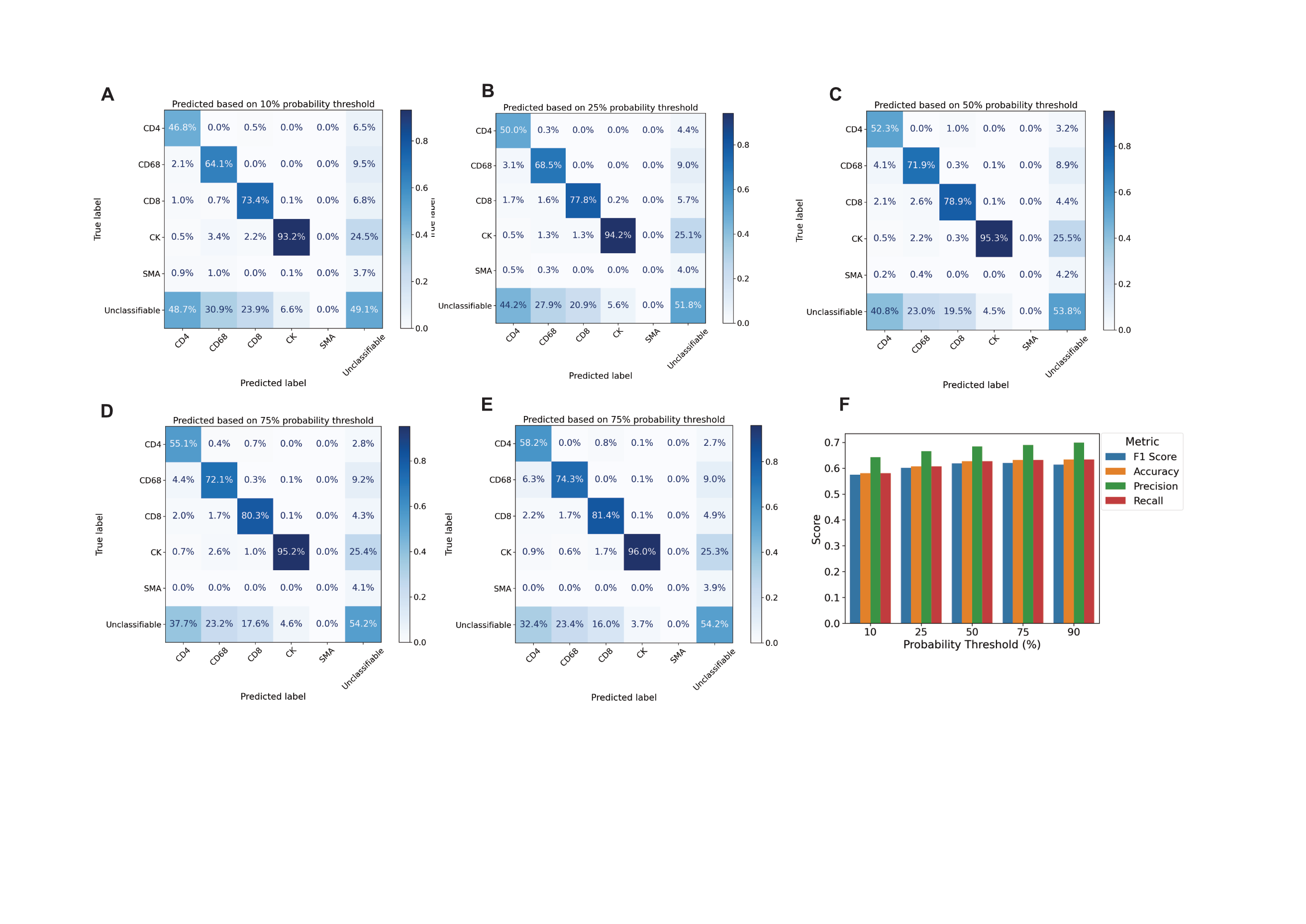

Supplement: S1 Fig — A-E. Confusion matrices comparing ground truth assignment of pan-cytokeratin, CD4, CD8, CD68, and SMA to PixlMap predictions by confidence thresholds. Percentages normalised to predications. F. Bar plot showing weighted performance statistics (F1 score, accuracy, precision, recall). (TIFF) [file pone.0317865.s001.tiff]
