# Supplementary material for: PixlMap: A generalisable pixel classifier for cellular phenotyping in multiplex immunofluorescence images
Source: PLoS One. 2025 Dec 3;20(12):e0317865. doi: 10.1371/journal.pone.0317865 (PMC12674528; doi:10.1371/journal.pone.0317865)
Supplement: S1 Table — (DOCX) [file pone.0317865.s002.docx]

|  | **Panel1 *** | | | | | | | | | |
| --- | --- | --- | --- | --- | --- | --- | --- | --- | --- | --- |
|  | **Reagent** | **Dilution** | **Incubation Time (min)** | **Temperature (°C)** | | **Supplier** | **Catalogue number** | | **Primary antibody lot number, where known** | **Primary antibody validation details**  Each primary antibody was run alongside a negative control on the tissue of interest to ensure secondary antibody specificity. |
|  | Bake (on board) | N/A | 32 | 60 | | N/A | N/A | |  | Robust validation from supplier including western blot in HeLa and Jurkat cells and IHC DAB validation in tonsil and Cleaved caspase-3 control cell pellets. Recommended parameters used for FFPE. Positive staining in necrotic regions of LUAD tissue assessed by an in-house pathologist. |
| **Cycle 1** | Discovery CC1 | RTU | 32 | 95 | | Roche Tissue Diagnostics | 950-500 | |  |  |
|  | Discovery Inhibitor | RTU | 12 | RT | | Roche Tissue Diagnostics | 760-4840 | |  |  |
|  | Cleaved caspase 3 (Asp175) | 1:200 | 32 | 37 | | Cell Signalling Technology | 9661 | | 47 |  |
|  | Omnimap anti-rabbit HRP | RTU | 12 | 37 | | Roche Tissue Diagnostics | 760-4311 | |  |  |
|  | Opal 650 | 1:200 | 8 | RT | | Akoya Biosciences | FP1496001KT | |  |  |
| **Cycle 2** | Discovery CC2 | RTU | 8 | 100 | | Roche Tissue Diagnostics | 950-123 | |  | Robust validation from supplier including western blot in HEK-293T cells. IHC DAB validation in human cardiac tissue, liver, and kidney from supplier. Recommended parameters used for FFPE. Positive staining assessed by an in-house pathologist in LUAD tissue. |
|  | Discovery Inhibitor | RTU | N/A | RT | | Roche Tissue Diagnostics | 760-4840 | |  |  |
|  | N-Cadherin (EPR1791-4) | 1:50 | 60 | 37 | | Abcam | 76011 | | GR3245174 |  |
|  | Omnimap anti-rabbit HRP | RTU | 32 | 37 | | Roche Tissue Diagnostics | 760-4311 | |  |  |
|  | Opal 520 | 1:100 | 8 | RT | | Akoya Biosciences | FP1487001KT | |  |  |
| **Cycle 3** | Discovery CC2 | RTU | 8 | 100 | | Roche Tissue Diagnostics | 950-123 | |  | Robust validation from supplier including IHC DAB in human tonsil. Recommended parameters used for FFPE. Positive endothelial cells in LUAD tissue assessed by an in-house pathologist. |
|  | Discovery Inhibitor | RTU | N/A | RT | | Roche Tissue Diagnostics | 760-4840 | |  |  |
|  | Goat Ig Block | RTU | 12 | RT | | Roche Tissue Diagnostics | 07988214001 | |  |  |
|  | Von Williebrand Factor (NCL-L-vWF) | 1:25 | 32 | 37 | | Leica Biosystems | NCL-L-vWF | |  |  |
|  | Omnimap anti-rabbit HRP | RTU | 16 | 37 | | Roche Tissue Diagnostics | 760-4311 | |  |  |
|  | Opal 570 | 1:100 | 8 | RT | | Akoya Biosciences | FP1488001KT | |  |  |
| **Cycle 4** | Discovery CC2 | RTU | 8 | 100 | | Roche Tissue Diagnostics | 950-123 | |  | Robust validation from supplier including western blot in H1299 cells and IHC validation in human ovarian carcinoma. Recommended parameters used for FFPE. Positive cytoplasmic staining in LUAD tissue assessed by an in-house pathologist. |
|  | Discovery Inhibitor | RTU | N/A | RT | | Roche Tissue Diagnostics | 760-4840 | |  |  |
|  | β-galactosidase (polyclonal) | 1:150 | 32 | 37 | | Abcam | 96239 | | GR3349352 |  |
|  | Omnimap anti-mouse HRP | RTU | 12 | 37 | | Roche Tissue Diagnostics | 760-4310 | |  |  |
|  | Opal 540 | 1:200 | 8 | RT | | Akoya Biosciences | FP1494001KT | |  |  |
| **Cycle 5** | Discovery CC2 | RTU | 8 | 100 | | Roche Tissue Diagnostics | 950-123 | |  | Robust validation from supplier including western blot in rat renal cortex lysates and in HeLa cells and Human Burkitt’s lymphoma cell line. IHC DAB validation from supplier in human breast cancer. Recommended parameters used for FFPE. Positive membranous staining in LUAD tissue assessed by an in-house pathologist. |
|  | Discovery Inhibitor | RTU | N/A | RT | | Roche Tissue Diagnostics | 760-4840 | |  |  |
|  | Goat Ig Block | RTU | 12 | RT | | Roche Tissue Diagnostics | 07988214001 | |  |  |
|  | Carbonic Anhydrase 9 (polyclonal) | 1:250 | 32 | 37 | | Abcam | 15086 | | GR32305793 |  |
|  | Omnimap anti-rabbit HRP | RTU | 12 | 37 | | Roche Tissue Diagnostics | 760-4311 | |  |  |
|  | Opal 690 | 1:200 | 8 | RT | | Akoya Biosciences | FP1497001KT | |  |  |
| **Cycle 6** | Discovery CC2 | RTU | 8 | 100 | | Roche Tissue Diagnostics | 950-123 | |  | Robust validation from the supplier including IHC DAB in human invasive ductal carcinoma. Recommended parameters used for FFPE. Positive epithelial cell staining in LUAD tissue assessed by an in-house pathologist. |
|  | Discovery Inhibitor | RTU | N/A | RT | | Roche Tissue Diagnostics | 760-4840 | |  |  |
|  | Pan-cytokeratin (AE1/AE3) | 1:250 | 28 | 37 | | Leica Biosystems | AE1/AE3-601-L-CE | | 608227 |  |
|  | Omnimap anti-mouse HRP | RTU | 12 | 37 | | Roche Tissue Diagnostics | 760-4310 | |  |  |
|  | Opal 620 | 1:100 | 8 | RT | | Akoya Biosciences | FP1495001KT | |  |  |
| **Cycle 7** | Discovery CC2 | RTU | 8 | 100 | | Roche Tissue Diagnostics | 950-123 | |  | Robust validation from supplier including western blot analysis and IHC DAB analysis to confirm specificity. Recommended parameters used for FFPE. Positive tumour cell membrane staining in LUAD tissue confirmed by an in-house pathologist. |
|  | Discovery Inhibitor | RTU | N/A | RT | | Roche Tissue Diagnostics | 760-4840 | |  |  |
|  | Protein cell death ligand 1 (E1L3N) | 1:50 | 120 | RT | | Cell Signalling Technology | 13684 | | 13 |  |
|  | Ultramap anti-rabbit HRP | RTU | 16 | 37 | | Roche Tissue Diagnostics | 05269717001 | |  |  |
|  | Opal 480 | 1:50 | 8 | RT | | Akoya Biosciences | FP1500001KT | |  |  |
| **Cycle 8** | Discovery CC2 | RTU | 8 | 100 | | Roche Tissue Diagnostics | 950-123 | |  | CE-IVD antibody approved. Robust validation from the supplier, recommended parameters used for FFPE. Positive nuclear staining in LUAD tissue assessed by an in-house pathologist. |
|  | Discovery Inhibitor | RTU | N/A | RT | | Roche Tissue Diagnostics | 760-4840 | |  |  |
|  | Goat Ig Block | RTU | 12 | RT | | Roche Tissue Diagnostics | 07988214001 | |  |  |
|  | Ki-67 (30-9) | RTU | 20 | 37 | | Roche Tissue Diagnostics | 05278384001 | | 6066456 |  |
|  | Omnimap anti-rabbit HRP | RTU | 12 | 37 | | Roche Tissue Diagnostics | 760-4311 | |  |  |
|  | TSA-DIG (manual application) | 1:100 | 24 | RT | | Akoya Biosciences | FP1502001KT | |  |  |
| **Cycle 9** | Opal 780 (manual application) | 1:10 | 60 | RT | | Akoya Biosciences | FP1501001KT | |  |  |
|  | QD DAPI (two drops) | RTU | 8 | RT | | Roche Tissue Diagnostics | 05268826001 | |  |  |
|  | **Panel 2 *** | | | | | | | | | |
|  | **Reagent** | **Dilution** | **Incubation Time (min)** | **Temperature (°C)** | | **Supplier** | **Catalogue number** | |  |  |
|  | Bake (on board) | N/A | 32 | 60 | | N/A | N/A | |  | Robust validation from supplier including IHC DAB using various tissue types and Jurkat (negative control) and THP-1 (positive control) cell pellets. Recommended parameters used for FFPE. Positive macrophage staining in human LUAD assessed by an in-house pathologist. |
| **Cycle 1** | Discovery CC1 | RTU | 64 | 95 | | Roche Tissue Diagnostics | 950-500 | |  | Antibody tested in IHC by supplier using various tissue types and Jurkat (negative control) and THP-1 (positive control) cell pellets. |
|  | Discovery Inhibitor | RTU | 12 | RT | | Roche Tissue Diagnostics | 760-4840 | |  |  |
|  | Goat Ig Block | RTU | 12 | RT | | Roche Tissue Diagnostics | 07988214001 | |  |  |
|  | CD68 (D4B9C) XP® | 1:200 | 32 | 37 | | Cell Signalling Technology | 76437 | | 4 |  |
|  | Omnimap anti-rabbit HRP | RTU | 16 | 37 | | Roche Tissue Diagnostics | 760-4311 | |  |  |
|  | Opal 690 | 1:100 | 8 | RT | | Akoya Biosciences | FP1497001KT | |  |  |
| **Cycle 2** | Discovery CC2 | RTU | 8 | 100 | | Roche Tissue Diagnostics | 950-123 | |  | Robust validation from the supplier including IHC DAB in human invasive ductal carcinoma. Recommended parameters used for FFPE. Positive epithelial cell staining in LUAD tissue assessed by an in-house pathologist. |
|  | Discovery Inhibitor | RTU | N/A | RT | | Roche Tissue Diagnostics | 760-4840 | |  |  |
|  | Pan-cytokeratin (AE1/AE3) | 1:250 | 28 | 37 | | Leica Biosystems | AE1/AE3-601-L-CE | | 6097192 |  |
|  | Omnimap anti-mouse HRP | RTU | 12 | 37 | | Roche Tissue Diagnostics | 760-4310 | |  |  |
|  | Opal 620 | 1:100 | 8 | RT | | Akoya Biosciences | FP1495001KT | |  |  |
| **Cycle 3** | Discovery CC2 | RTU | 8 | 100 | | Roche Tissue Diagnostics | 950-123 | |  | CE-IVD antibody approved. Robust validation from the supplier, recommended parameters used for FFPE. Positive endothelial cell and cytoplasmic staining in LUAD tissue assessed by an in-house pathologist. |
|  | Discovery Inhibitor | RTU | N/A | RT | | Roche Tissue Diagnostics | 760-4840 | |  |  |
|  | Goat Ig Block | RTU | 24 | RT | | Roche Tissue Diagnostics | 07988214001 | |  |  |
|  | Smooth Muscle Actin (1A4) | RTU | 32 | 37 | | Roche Tissue Diagnostics | 760-2833 | | V0002130 |  |
|  | Omnimap anti-mouse HRP | RTU | 12 | 37 | | Roche Tissue Diagnostics | 760-4310 | |  |  |
|  | Opal 540 | 1:200 | 8 | RT | | Akoya Biosciences | FP1494001KT | |  |  |
| **Cycle 4** | Discovery CC2 | RTU | 8 | 100 | | Roche Tissue Diagnostics | 950-123 | |  | CE-IVD antibody approved. Robust validation from the supplier, recommended parameters used for FFPE. Positive T cell staining in tonsil and human LUAD were reviewed by an in-house pathologist. |
|  | Discovery Inhibitor | RTU | N/A | RT | | Roche Tissue Diagnostics | 760-4840 | |  |  |
|  | Goat Ig Block | RTU | 24 | RT | | Roche Tissue Diagnostics | 07988214001 | |  |  |
|  | CD4 (SP35) | RTU | 60 | 37 | | Roche Tissue Diagnostics | 790-4423 | | H23372 |  |
|  | Omnimap anti-rabbit HRP | RTU | 16 | 37 | | Roche Tissue Diagnostics | 760-4311 | |  |  |
|  | Opal 570 | 1:50 | 8 | RT | | Akoya Biosciences | FP1488001KT | |  |  |
| **Cycle 5** | Discovery CC2 | RTU | 8 | 100 | | Roche Tissue Diagnostics | 950-123 | |  | Robust validation from the supplier. Antibody validated for IHC DAB in tonsil and then the tissue of interest in-house. Recommended parameters used for FFPE. Positive control and T cell staining reviewed by an in-house pathologist. |
|  | Discovery Inhibitor | RTU | N/A | RT | | Roche Tissue Diagnostics | 760-4840 | |  |  |
|  | CD8 α (C8/144B) | 1:100 | 56 | 37 | | Cell Signalling Technology | 70306 | | 1 |  |
|  | Omnimap anti-mouse HRP | RTU | 12 | 37 | | Roche Tissue Diagnostics | 760-4310 | |  |  |
|  | Opal 520 | 1:100 | 8 | RT | | Akoya Biosciences | FP1487001KT | |  |  |
| **Cycle 6** | Discovery CC2 | RTU | 8 | 100 | | Roche Tissue Diagnostics | 950-123 | |  | CE-IVD antibody approved. Robust validation from the supplier, recommended parameters used for FFPE. Positive nuclear staining in LUAD tissue assessed by an in-house pathologist. |
|  | Discovery Inhibitor | RTU | N/A | RT | | Roche Tissue Diagnostics | 760-4840 | |  |  |
|  | Goat Ig Block | RTU | 24 | RT | | Roche Tissue Diagnostics | 07988214001 | |  |  |
|  | Ki-67 (30-9) | RTU | 20 | 37 | | Roche Tissue Diagnostics | 05278384001 | | H23606 |  |
|  | Omnimap anti-rabbit HRP | RTU | 12 | 37 | | Roche Tissue Diagnostics | 760-4311 | |  |  |
|  | Opal 650 | 1:400 | 8 | RT | | Akoya Biosciences | FP1496001KT | |  |  |
|  | QD DAPI (two drops) | RTU | 24 | RT | | Roche Tissue Diagnostics | 05268826001 | |  |  |
|  | **Panel 3 part 1*** | | | | | | | | | |
|  | Bake (on board) | N/A | 32 | 60 | | N/A | N/A | |  | Robust validation from supplier including western blot analysis in multiple cell lines. Validated in IHC DAB by supplier in various tissue types. Recommended parameters used for FFPE. Positive nuclear staining assessed by an in-house pathologist in LUAD tissue. |
| **Cycle 1** | Discovery CC1 | RTU | 64 | 95 | | Roche Tissue Diagnostics | 950-500 | |  |  |
|  | Discovery Inhibitor | RTU | 12 | RT | | Roche Tissue Diagnostics | 760-4840 | |  |  |
|  | Activating Transcription Factor 4 (EPR18111) | 1:25 | 120 | RT | | Abcam | ab184909 | | GR3360041 |  |
|  | Goat Ig Block | RTU | 20 | RT | | Roche Tissue Diagnostics | 07988214001 | |  |  |
|  | Ultramap anti-rabbit HRP | RTU | 24 | 37 | | Roche Tissue Diagnostics | 05269717001 | |  |  |
|  | Opal 480 | 1:25 | 8 | RT | | Akoya Biosciences | FP1500001KT | |  |  |
| **Cycle 2** | Discovery CC2 | RTU | 8 | 100 | | Roche Tissue Diagnostics | 950-123 | |  | Robust validation from supplier including western blot analysis in MCF7 and NIH/3T3 cells and in starved MCF-7 cells. IHC DAB validation from supplier in human colon carcinoma and human lung carcinoma and positive and negative control cell pellets. Recommended parameters used for FFPE. Positive staining assessed by an in-house pathologist in LUAD tissue. |
|  | Discovery Inhibitor | RTU | N/A | RT | | Roche Tissue Diagnostics | 760-4840 | |  |  |
|  | Phosphorylated S6 Ribosomal Protein (Ser240/244) (D68F8) | 1:200 | 60 | 37 | | Cell Signalling Technology | 5364 | | 8 |  |
|  | Ultramap anti-rabbit HRP | RTU | 20 | 37 | | Roche Tissue Diagnostics | 05269717001 | |  |  |
|  | Opal 520 | 1:400 | 8 | RT | | Akoya Biosciences | FP1487001KT | |  |  |
| **Cycle 3** | Discovery CC2 | RTU | 8 | 100 | | Roche Tissue Diagnostics | 950-123 | |  | Robust validation from supplier including western blot analysis from various cell lines including HeLa cells. IHC DAB validation from supplier in human lung carcinoma. Recommended parameters used for FFPE. Positive staining assessed in LUAD tissue by an in-house pathologist. |
|  | Discovery Inhibitor | RTU | N/A | RT | | Roche Tissue Diagnostics | 760-4840 | |  |  |
|  | e-IF2α (D7D3) XP® | 1:2500 | 32 | 37 | | Cell Signalling Technology | 5324 | | 9 |  |
|  | Omnimap anti-rabbit HRP | RTU | 12 | 37 | | Roche Tissue Diagnostics | 760-4311 | |  |  |
|  | Opal 650 | 1:100 | 8 | RT | | Akoya Biosciences | FP1496001KT | |  |  |
| **Cycle 4** | Discovery CC2 | RTU | 8 | 100 | | Roche Tissue Diagnostics | 950-123 | |  | Robust validation from supplier including western blot analysis in C2C12 cells and IHC DAB validation in human colon and human lung carcinoma and human lymphoma. Recommended parameters used for FFPE. Positive staining in LUAD tissue assessed by an in-house pathologist. |
|  | Discovery Inhibitor | RTU | N/A | RT | | Roche Tissue Diagnostics | 760-4840 | |  |  |
|  | p-eIF2α (Ser51) (D9G8) XP® | 1:25 | 6(hours) | RT | | Cell Signalling Technology | 3398 | | 6 |  |
|  | Ultramap anti-rabbit HRP | RTU | 28 | 37 | | Roche Tissue Diagnostics | 05269717001 | |  |  |
|  | Opal 570 | 1:100 | 8 | RT | | Akoya Biosciences | FP1488001KT | |  |  |
|  | **Panel 3 part 2** | | | | | | | | | |
|  | Wash slide with Reaction Buffer x3 for 1 min and place slide back on Ventana Discovery Ultra with part 2 label with part 2 reagents. Cover slide in reaction buffer and run machine. | | | | | | | | | |
| **Cycle 5 (wet slide load)** | Discovery CC2 | RTU | 8 | 100 | | Roche Tissue Diagnostics | 950-123 | |  |  |
|  | Discovery Inhibitor | RTU | N/A | RT | | Roche Tissue Diagnostics | 760-4840 | |  | Robust validation from the supplier including IHC DAB in human invasive ductal carcinoma. Recommended parameters used for FFPE. Positive epithelial cell staining in LUAD tissue assessed by an in-house pathologist. |
|  | Pan-cytokeratin (AE1/AE3) | 1:250 | 28 | 37 | | Leica Biosystems | AE1/AE3-601-L-CE | | 6082227 |  |
|  | Omnimap anti-mouse HRP | RTU | 12 | 37 | | Roche Tissue Diagnostics | 760-4310 | |  |  |
|  | Opal 620 | 1:100 | 8 | RT | | Akoya Biosciences | FP1495001KT | |  |  |
| **Cycle 6** | Discovery CC2 | RTU | 8 | 100 | | Roche Tissue Diagnostics | 950-123 | |  | Robust validation from supplier including western blot analysis from various cell lines including HeLa, NIH/3T3, PC12, and COS cells. IHC DAB validation from supplier on human breast, lung, and prostate carcinoma and in Non-Hodgkin lymphoma. Recommended parameters used for FFPE. Positive staining in LUAD tissue assessed by an in-house pathologist. |
|  | Discovery Inhibitor | RTU | N/A | RT | | Roche Tissue Diagnostics | 760-4840 | |  |  |
|  | Ribosomal Protein S6 (5G10) | 1:50 | 60 | 37 | | Cell Signalling Technology | 2217 | | 10 |  |
|  | Ultramap anti-rabbit HRP | RTU | 12 | 37 | | Roche Tissue Diagnostics | 05269717001 | |  |  |
|  | Opal 690 | 1:100 | 8 | RT | | Akoya Biosciences | FP1497001KT | |  |  |
| **Cycle 7** | Discovery CC2 | RTU | 8 | 100 | | Roche Tissue Diagnostics | 950-123 | |  | Robust validation from supplier including western blot analysis in 293T cells. IHC DAB validation from supplier in human colon carcinoma, human lymphoma, and positive and negative control cell pellets. Recommended parameters used for FFPE. Positive staining in LUAD tissue assessed by an in-house pathologist. |
|  | Discovery Inhibitor | RTU | N/A | RT | | Roche Tissue Diagnostics | 760-4840 | |  |  |
|  | Phosphorylated ErbB3-binding protein 1 (Thr37/46) (236B4) | 1:50 | 60 | 37 | | Cell Signalling Technology | 2855 | | 26 |  |
|  | Omnimap anti-rabbit HRP | RTU | 12 | 37 | | Roche Tissue Diagnostics | 760-4311 | |  |  |
|  | Opal 540 | 1:200 | 8 | RT | | Akoya Biosciences | FP1494001KT | |  |  |
| **Cycle 8** | Discovery CC2 | RTU | 8 | 100 | | Roche Tissue Diagnostics | 950-123 | |  | CE-IVD antibody approved. Robust validation from the supplier, recommended parameters used for FFPE. Positive nuclear staining in LUAD tissue assessed by an in-house pathologist. |
|  | Discovery Inhibitor | RTU | N/A | RT | | Roche Tissue Diagnostics | 760-4840 | |  |  |
|  | Goat Ig Block | RTU | 24 | RT | | Roche Tissue Diagnostics | 07988214001 | |  |  |
|  | Ki-67 (30-9) | RTU | 20 | 37 | | Roche Tissue Diagnostics | 05278384001 | | G08030 |  |
|  | Omnimap anti-rabbit HRP | RTU | 12 | 37 | | Roche Tissue Diagnostics | 760-4311 | |  |  |
|  | TSA-DIG (manual application) | 1:100 | 24 | RT | | Akoya Biosciences | FP1502001KT | |  |  |
| **Cycle 9** | Opal 780 (manual application) | 1:10 | 60 | RT | | Akoya Biosciences | FP1501001KT | |  |  |
|  | QD DAPI (two drops) | RTU | 12 | RT | | Roche Tissue Diagnostics | 05268826001 | |  |  |
|  | **Panel 4 *** | | | | | | | | | |
| **Cycle 1** | Bake (on board) | N/A | 32 | 60 | | N/A | N/A | |  |  |
|  | Discovery CC1 | RTU | 64 | 95 | | Roche Tissue Diagnostics | 950-123 | |  | Robust validation from supplier including IHC DAB using various tissue types and Jurkat (negative control) and THP-1 (positive control) cell pellets. Recommended parameters used for FFPE. Positive macrophage staining in human LUAD assessed by an in-house pathologist. |
|  | Discovery Inhibitor | RTU | N/A | RT | | Roche Tissue Diagnostics | 760-4840 | |  |  |
|  | Goat Ig Block | RTU | 24 | RT | | Roche Tissue Diagnostics | 07988214001 | |  |  |
|  | CD68 (D4B9C) XP® | 1:200 | 32 | 37 | | Cell Signalling Technology | 76437 | | 76437S |  |
|  | Omnimap anti-rabbit HRP | RTU | 16 | 37 | | Roche Tissue Diagnostics | 760-4311 | |  |  |
|  | Opal 620 | 1:100 | 8 | RT | | Akoya Biosciences | FP1495001KT | |  |  |
| **Cycle 2** | Discovery CC2 | RTU | 8 | 100 | | Roche Tissue Diagnostics | 950-123 | |  | Advanced validation from supplier including western blot analysis in HEK-293. IHC DAB validation from supplier in various tissue types. Recommended parameters used for FFPE for validation in tonsil and then LUAD. Positive T cell staining in human tonsil and LUAD tissue assessed by an in-house pathologist. |
|  | Discovery Inhibitor | RTU | N/A | RT | | Roche Tissue Diagnostics | 760-4840 | |  |  |
|  | FOXP3 | 1:20 | 32 | 37 | | Abcam | 20034 | | 1 |  |
|  | Omnimap anti-mouse HRP | RTU | 12 | 37 | | Roche Tissue Diagnostics | 760-4310 | |  |  |
|  | Opal 690 | 1:200 | 8 | RT | | Akoya Biosciences | FP1497001KT | |  |  |
| **Cycle 3** | Discovery CC2 | RTU | 8 | 100 | | Roche Tissue Diagnostics | 950-123 | |  | CE-IVD antibody approved. Robust validation from the supplier. Recommended parameters used for FFPE. Positive B cell staining in tonsil and then LUAD assessed by an in-house pathologist. |
|  | Discovery Inhibitor | RTU | N/A | RT | | Roche Tissue Diagnostics | 760-4840 | |  |  |
|  | CD20 | RTU | 32 | 37 | | Roche Tissue Diagnostics | 760-2531 | | F17178 |  |
|  | Omnimap anti-mouse HRP | RTU | 12 | 37 | | Roche Tissue Diagnostics | 760-4310 | |  |  |
|  | Opal 540 | 1:300 | 8 | RT | | Akoya Biosciences | FP1494001KT | |  |  |
| **Cycle 4** | Discovery CC2 | RTU | 8 | 100 | | Roche Tissue Diagnostics | 950-123 | |  | CE-IVD antibody approved. Robust validation from the supplier. Recommended parameters used for FFPE. Positive T cell staining in tonsil and then LUAD assessed by an in-house pathologist. |
|  | Discovery Inhibitor | RTU | N/A | RT | | Roche Tissue Diagnostics | 760-4840 | |  |  |
|  | CD4 (SP35) | RTU | 32 | 37 | | Roche Tissue Diagnostics | 790-4423 | | F20053 |  |
|  | Omnimap anti-rabbit HRP | RTU | 16 | 37 | | Roche Tissue Diagnostics | 760-4311 | |  |  |
|  | Opal 570 | 1:200 | 8 | RT | | Akoya Biosciences | FP1488001KT | |  |  |
| **Cycle 5** | Discovery CC2 | RTU | 8 | 100 | | Roche Tissue Diagnostics | 950-123 | |  | Robust validation from the supplier. Antibody validated for IHC DAB in tonsil and then the tissue of interest in-house. Positive control and T cell staining in human tonsil and LUAD reviewed by an in-house pathologist. |
|  | Discovery Inhibitor | RTU | N/A | RT | | Roche Tissue Diagnostics | 760-4840 | |  |  |
|  | CD8 α (C8/144B) | 1:100 | 56 | 37 | | Cell Signalling Technology | 70306 | | 1 |  |
|  | Omnimap anti-mouse HRP | RTU | 12 | 37 | | Roche Tissue Diagnostics | 760-4310 | |  |  |
|  | Opal 520 | 1:100 | 8 | RT | | Akoya Biosciences | FP1487001KT | |  |  |
| **Cycle 6** | Discovery CC2 | RTU | 8 | 100 | | Roche Tissue Diagnostics | 950-123 | |  | Robust validation from the supplier including IHC DAB in human invasive ductal carcinoma. Recommended parameters used. Positive epithelial cell staining in LUAD tissue assessed by an in-house pathologist. |
|  | Discovery Inhibitor | RTU | N/A | RT | | Roche Tissue Diagnostics | 760-4840 | |  |  |
|  | Pan-cytokeratin (AE1/AE3) | 1:250 | 28 | 37 | | Leica Biosystems | AE1/AE3-601-L-CE | | 6066984 |  |
|  | Omnimap anti-mouse HRP | RTU | 12 | 37 | | Roche Tissue Diagnostics | 760-4310 | |  |  |
|  | Opal 620 | 1:100 | 8 | RT | | Akoya Biosciences | FP1495001KT | |  |  |
|  | QD DAPI (two drops) | RTU | 12 | RT | | Roche Tissue Diagnostics | 05268826001 | |  |  |
|  | **Panel 5** | | | | | | | | | |
| **Cycle 1** | Bake (on board) | N/A | 32 | 60 | | N/A | N/A | |  | Positive control (mouse spleen) was used to validate the assay and double positivity with CD8 was assessed by an in-house pathologist in mouse liver tissue. |
|  | Discovery CC1 | RTU | 64 | 95 | | Roche Tissue Diagnostics | 950-123 | |  |  |
|  | Discovery Inhibitor | RTU | N/A | RT | | Roche Tissue Diagnostics | 760-4840 | |  |  |
|  | GranzymeB (polyclonal) | 1:100 | 32 | 37 | | Novus Biologicals | NB100-684 | | R705 | Positive control (mouse spleen) was used to validate the assay and double positivity with CD8 was assessed by an in-house pathologist in Ms liver. |
|  | Omnimap anti-rabbit HRP | RTU | 12 | 37 | | Roche Tissue Diagnostics | 760-4311 | |  |  |
|  | Opal 650 | 1:300 | 8 | RT | | Akoya Biosciences | FP1496001KT | |  |  |
| **Cycle 2** | Discovery CC2 | RTU | 8 | 100 | | Roche Tissue Diagnostics | 950-123 | |  | Relative expression by manufacturer. Recommended parameters used. Positive T cell staining in mouse spleen and mouse liver tissue assessed by an in-house pathologist. |
|  | Discovery Inhibitor | RTU | N/A | RT | | Roche Tissue Diagnostics | 760-4840 | |  |  |
|  | CD8 (4SM15) | 1:75 | 32 | RT | | ThermoFisher Scientific | 14-0808-82 | | 2720194 | Relative expression by manufacturer. |
|  | Omnimap anti-rat HRP | RTU | 20 | RT | | Roche Tissue Diagnostics | 760-4457 | |  |  |
|  | Opal 690 | 1:200 | 8 | RT | | Akoya Biosciences | FP1497001KT | |  |  |
| **Cycle 3** | Discovery CC2 | RTU | 8 | 100 | | Roche Tissue Diagnostics | 950-123 | |  | CE-IVD antibody approved. Robust validation from supplier. A positive control (Human tonsil) and mouse liver tissue staining was assessed by an in-house pathologist. |
|  | Discovery Inhibitor | RTU | N/A | RT | | Roche Tissue Diagnostics | 760-4840 | |  |  |
|  | c-MYC (Y69) | RTU | 4 (hours) | RT | | Roche Tissue Diagnostics | 790-4628 | | K05962 | CE-IVD antibody approved, a positive control (Human tonsil) was used to validate the assay and Ms liver staining was assessed by an in-house pathologist. |
|  | Anti-Rabbit HQ | RTU | 60 | RT | | Roche Tissue Diagnostics | 760-4815 | |  |  |
|  | Anti-HQ HRP | RTU | 32 | RT | | Roche Tissue Diagnostics | 07017936001 | |  |  |
|  | Opal 570 | 1:150 | 8 | RT | | Akoya Biosciences | FP1488001KT | |  |  |
| **Cycle 4** | Discovery CC2 | RTU | 8 | 100 | | Roche Tissue Diagnostics | 950-123 | |  | Antibody tested in western blot and IHC by supplier. Positive T cell staining in mouse spleen and mouse liver tissue were assessed by an in-house pathologist. |
|  | Discovery Inhibitor | RTU | N/A | RT | | Roche Tissue Diagnostics | 760-4840 | |  |  |
|  | Goat Ig Block | RTU | 24 | RT | | Roche Tissue Diagnostics | 07988214001 | |  |  |
|  | CD45 (polyclonal) | 1:300 | 32 | 37 | | Abcam | Ab10558 | | 1041690-2 | Antibody tested in WB and IHC by supplier, immune cells were assessed by an in-house pathologist. |
|  | Omnimap anti-rat HRP | RTU | 20 | RT | | Roche Tissue Diagnostics | 760-4457 | |  |  |
|  | Opal 480 | 1:50 | 8 | RT | | Akoya Biosciences | FP1500001KT | |  |  |
| **Cycle 5** | Discovery CC2 | RTU | 8 | 100 | | Roche Tissue Diagnostics | 950-123 | |  | Cell treatment by manufacturer. Robust validation by supplier. Recommended parameters used for FFPE. Immune cells and sinusoids in mouse liver were assessed by an in-house pathologist. |
|  | Discovery Inhibitor | RTU | N/A | RT | | Roche Tissue Diagnostics | 760-4840 | |  |  |
|  | CD4 (4SM15) | 1:25 | 60 | 37 | | ThermoFisher Scientific | 14-9766 | | 2526300 | cell treatment by manufacturer, immune cells in mouse liver were assessed by an in-house pathologist. |
|  | Impress rat | RTU | 48 | RT | | Vector Laboratories | ZJ0512 | |  |  |
|  | Opal 620 | 1:50 | 8 | RT | | Akoya Biosciences | FP1495001KT | |  |  |
| **Cycle 6** | Discovery CC2 | RTU | 8 | 100 | | Roche Tissue Diagnostics | 950-123 | |  | Robust validation from the supplier including IHC DAB in mouse metastatic tumour tissue, mouse thymus, and mouse spleen. Recommended parameters used for FFPE. Positive T cell staining in mouse spleen and mouse liver assessed by an in-house pathologist. |
|  | Discovery Inhibitor | RTU | N/A | RT | | Roche Tissue Diagnostics | 760-4840 | |  |  |
|  | FOXP3 (D6O8R) | 1:50 | 32 | 37 | | Cell Signalling Technology | 12653 | | 9 |  |
|  | Omnimap anti-rabbit HRP | RTU | 12 | 37 | | Roche Tissue Diagnostics | 760-4311 | |  |  |
|  | Opal 540 | 1:100 | 8 | RT | | Akoya Biosciences | FP1494001KT | |  |  |
| **Cycle 7** | Discovery CC2 | RTU | 8 | 100 | | Roche Tissue Diagnostics | 950-123 | |  | Antibody tested in western blot and IHC by supplier, zonal specificity in liver by researchers. Recommended parameters used for FFPE. GS positivity assessed by an in-house pathologist. |
|  | Discovery Inhibitor | RTU | N/A | RT | | Roche Tissue Diagnostics | 760-4840 | |  |  |
|  | GS (polyclonal) | 1:500 | 32 | 37 | | Sigma Aldrich | HPA007316 | | 16878 | Antibody tested in WB and IHC by supplier, zonal specificity in liver by researchers. Positive staining assessed by an in-house pathologist on mouse liver tissue. |
|  | Omnimap anti-rabbit HRP | RTU | 12 | 37 | | Roche Tissue Diagnostics | 760-4311 | |  |  |
|  | Opal 520 | 1:100 | 8 | RT | | Akoya Biosciences | FP1487001KT | |  |  |
| **Cycle 8** | Discovery CC2 | RTU | 8 | 100 | | Roche Tissue Diagnostics | 950-123 | |  | Antibody validated by supplier with western blot analysis in HeLa Cells and IHC DAB in various tissue types. Recommended parameters used in FFPE. Zonal specificity in liver assessed by an in-house pathologist. |
|  | Discovery Inhibitor | RTU | N/A | RT | | Roche Tissue Diagnostics | 760-4840 | |  |  |
|  | B-catenin (D10A8) | 1:25 | 2 | RT | | Cell Signalling Technology | 8480 | | 9 | Antibody tested by supplier with HeLa Cells and tissue, zonal specificity in liver assessed by an in-house pathologist. |
|  | QD DAPI (three drops) | RTU | 24 | RT | | Roche Tissue Diagnostics | 05268826001 | |  |  |
|  | **Panel 6** | | | | | | | | | |
| **Cycle 1** | Bake (on board) | N/A | 32 | 60 | | N/A | N/A | |  | CE-IVD antibody approved. Robust validation from supplier. Recommended parameters used. Positive cytoplasmic fibroblast staining assessed by an in-house pathologist in LUAD tissue. |
|  | Discovery CC1 | RTU | 64 | 95 | | Roche Tissue Diagnostics | 950-123 | |  |  |
|  | Discovery Inhibitor | RTU | N/A | RT | | Roche Tissue Diagnostics | 760-4840 | |  |  |
|  | Podoplanin (D2-40) | RTU | 32 | 37 | | Roche Tissue Diagnostics | 760-4395 | | V0004499 |  |
|  | Omnimap anti-mouse HRP | RTU | 24 | RT | | Roche Tissue Diagnostics | 760-4310 | |  |  |
|  | Discovery Cy5 | RTU | 12 | RT | | Roche Tissue Diagnostics | 760-238 | |  |  |
| **Cycle 2** | Discovery CC2 | RTU | 8 | 100 | | Roche Tissue Diagnostics | 950-123 | |  | Robust validation from supplier including western blot analysis on Raji, Daudi, and HEK-293 whole cell lysates. IHC DAB validation from supplier using various tissue types. Recommended parameters used. Positive fibroblast staining in LUAD assessed by an in-house pathologist. |
|  | Discovery Inhibitor | RTU | N/A | RT | | Roche Tissue Diagnostics | 760-4840 | |  |  |
|  | Anti-HLA-DR (TAL-1B5) | 1:500 | 32 | 37 | | Abcam | Ab20181 | | 1003483-7 |  |
|  | Omnimap anti-mouse HRP | RTU | 12 | RT | | Roche Tissue Diagnostics | 760-4310 | |  |  |
|  | Discovery RED610 | RTU | 60 | RT | | Roche Tissue Diagnostics | 760-245 | |  |  |
| **Cycle 3** | Discovery CC2 | RTU | 8 | 100 | | Roche Tissue Diagnostics | 950-123 | |  | CE-IVD antibody approved. Robust validation from the supplier. Recommended parameters used. Positive endothelial cell and cytoplasmic fibroblast staining in LUAD tissue assessed by an in-house pathologist. |
|  | Discovery Inhibitor | RTU | N/A | RT | | Roche Tissue Diagnostics | 760-4840 | |  |  |
|  | Goat Ig Block | RTU | 12 | RT | | Roche Tissue Diagnostics | 07988214001 | |  |  |
|  | Actin, Smooth Muscle (1A4) | RTU | 16 | 37 | | Roche Tissue Diagnostics | 5268303001 | | V0003629 |  |
|  | Omnimap anti-mouse HRP | RTU | 8 | RT | | Roche Tissue Diagnostics | 760-4310 | |  |  |
|  | Discovery DCC | TRU | 4 | RT | | Roche Tissue Diagnostics | 760-240 | |  |  |
| **Cycle 4** | Discovery CC2 | RTU | 8 | 100 | | Roche Tissue Diagnostics | 950-123 | |  |  |
|  | Discovery Inhibitor | RTU | N/A | RT | | Roche Tissue Diagnostics | 760-4840 | |  | Robust validation from supplier including western blot analysis including human spleen, human lung, and rat lung cell lines. Recommended parameters used. Fibroblast staining assessed in LUAD tissue by an in-house pathologist. |
|  | Goat Ig Block | RTU | 12 | RT | | Roche Tissue Diagnostics | 07988214001 | |  |  |
|  | Interleukin 6 (1.2-2B11-2G10) | 1:25 | 60 | 37 | | Abcam | Ab9324 | | 1021638-1 |  |
|  | Omnimap anti-mouse HRP | RTU | 12 | RT | | Roche Tissue Diagnostics | 760-4310 | |  |  |
|  | Discovery FAM | RTU | 8 | RT | | Roche Tissue Diagnostics | 760-243 | |  |  |
| **Cycle 5** | Discovery CC2 | RTU | 8 | 100 | | Roche Tissue Diagnostics | 950-123 | |  | Robust validation from the supplier including western blot analysis from various cell lines. Confirmed low expression consistency with literature in SK-MEL-28 cells. IHC DAB analysis from supplier with various tissue types. Recommended parameters used. Positive fibroblast staining in LUAD assessed by an in-house pathologist. |
|  | Discovery Inhibitor | RTU | N/A | RT | | Roche Tissue Diagnostics | 760-4840 | |  |  |
|  | Fibroblast Activation Protein (F1A4G) | 1:25 | 60 | 37 | | Cell Signalling Technology | 528185 | | 1 |  |
|  | Omnimap anti-rabbit HRP | RTU | 12 | 37 | | Roche Tissue Diagnostics | 760-4311 | |  |  |
|  | Discovery Rhodamine 6G | RTU | 20 | RT | | Roche Tissue Diagnostics | 760-244 | |  |  |
| **Cycle 6** | Discovery CC2 | RTU | 8 | 100 | | 950-123 |  | |  | Robust validation from the supplier including IHC DAB in human invasive ductal carcinoma. Recommended parameters used. Positive epithelial cell staining assessed by an in-house pathologist in LUAD tissue. |
|  | Discovery Inhibitor | RTU | N/A | RT | | 760-4840 |  | |  |  |
|  | Pan-Cytokeratin (AE1/AE3) | 1:250 | 28 | 37 | | Leica Biosciences | NCL-L-AE1/AE3-601-L-CE | | 6116099 |  |
|  | Omnimap anti-mouse HRP | RTU | 12 | RT | | Roche Tissue Diagnostics | 760-4310 | |  |  |
|  | TSA-DIG (manual application) | 1:100 | 20 | RT | | Akoya Biosciences | FP1502001KT | |  |  |
| **Cycle 7** | Opal 780 (manual application) | 1:10 | 60 | RT | | Akoya Biosciences | FP1501001KT | |  |  |
|  | QD DAPI (two drops) | RTU | 8 | RT | | Roche Tissue Diagnostics | 05268826001 | |  |  |
|  | **Panel 7** | | | | | | | | | |
| **Cycle 1** | Bake (on board) | N/A | 32 | 60 | | N/A | N/A | |  | Robust validation from the supplier with western blot and IHC in human Kidney FFPE.  IHC optimisation in-house on the positive control suggested from the supplier, specificity assessed by an in-house pathologist in LUAD tissue. |
|  | Discovery CC1 | RTU | 64 | 95 | | Roche Tissue Diagnostics | 950-123 | |  |  |
|  | Discovery Inhibitor | RTU | N/A | RT | | Roche Tissue Diagnostics | 760-4840 | |  |  |
|  | Goat Ig Block | RTU | 12 | RT | | Roche Tissue Diagnostics | 07988214001 | |  |  |
|  | ATP5a (EPR13030(B)) | 1:250 | 32 | 37 | | Abcam | AB176569 | | 1014592-3 |  |
|  | Omnimap anti-rabbit HRP | RTU | 12 | 37 | | Roche Tissue Diagnostics | 760-4311 | |  |  |
|  | Opal 690 | 1:300 | 8 | RT | | Akoya Biosciences | FP1497001KT | |  |  |
| **Cycle 2** | Discovery CC2 | RTU | 8 | 100 | | Roche Tissue Diagnostics | 950-123 | |  | Robust validation from the supplier with western blot and IHC.  IHC optimisation in house on human Colorectal cancer and normal colon. Overexpression of nucleoli on Colorectal cancer and LUAD tissue assessed by an in-house pathologist. |
|  | Discovery Inhibitor | RTU | N/A | RT | | Roche Tissue Diagnostics | 760-4840 | |  |  |
|  | Goat Ig Block | RTU | 12 | RT | | Roche Tissue Diagnostics | 07988214001 | |  |  |
|  | UBF1 (ep2741Y) | 1:50 | 32 | 37 | | Abcam | Ab75781 | | GR103297-S |  |
|  | Omnimap anti-rabbit HRP | RTU | 12 | 37 | | Roche Tissue Diagnostics | 760-4311 | |  |  |
|  | Opal 520 | 1:100 | 8 | RT | | Akoya Biosciences | FP1487001KT | |  |  |
| **Cycle 3** | Discovery CC2 | RTU | 8 | 100 | | Roche Tissue Diagnostics | 950-123 | |  | Antibody validated on IHC by the supplier. Internal assessment of cellular compartment positivity by an in-house pathologist in the LUAD tissue. |
|  | Discovery Inhibitor | RTU | N/A | RT | | Roche Tissue Diagnostics | 760-4840 | |  |  |
|  | GOLGB1 (polyclonal) | 1:300 | 60 | 37 | | Sigma-Aldrich | HPA011008 | | A80309 |  |
|  | Omnimap anti-rabbit HRP | RTU | 12 | 37 | | Roche Tissue Diagnostics | 760-4311 | |  |  |
|  | Opal 650 | 1:300 | 8 | RT | | Akoya Biosciences | FP1496001KT | |  |  |
| **Cycle 4** | Discovery CC2 | RTU | 8 | 100 | | Roche Tissue Diagnostics | 950-123 | |  | Antibody KO/KD validated by the supplier. IHC validated in house and membrane staining assessed by an in-house pathologist in LUAD tissue. |
|  | Discovery Inhibitor | RTU | N/A | RT | | Roche Tissue Diagnostics | 760-4840 | |  |  |
|  | Goat Ig Block | RTU | 12 | RT | | Roche Tissue Diagnostics | 07988214001 | |  |  |
|  | CD98/SLC3A2 (polyclonal) | 1:100 | 32 | 37 | | Protein Tech Laboratories | 15193-1-AP | | *00090199 |  |
|  | Goat Ig Block | RTU | 12 | RT | | Roche Tissue Diagnostics | 07988214001 | |  |  |
|  | Sodium Potassium ATPase Alpha 1 (464.6) | 1:50 | 32 | 32 | | Novus Biologicals | NB300-146 | | C-2 | Robust validation by the supplier with western blot and IHC.  Kidney cells expression level assessed by an in-house pathologist after in-house validation on LUAD tissue. |
| **Cycle5** | Omnimap anti-rabbit HRP | RTU | 16 | 37 | | Roche Tissue Diagnostics | 760-4311 | |  |  |
|  | Opal 620 | 1:100 | 8 | RT | | Akoya Biosciences | FP1495001KT | |  |  |
| **Cycle 6** | Discovery CC2 | RTU | 8 | 100 | | Roche Tissue Diagnostics | 950-123 | |  | Antibody validated by the supplier both in Western Blot and IHC. In house optimisation, cytoplasm staining assessed by an in-house pathologist in LUAD tissue. |
|  | Discovery Inhibitor | RTU | N/A | RT | | Roche Tissue Diagnostics | 760-4840 | |  |  |
|  | Calnexin (C5C9) | 1:50 | 32 | 37 | | Cell Signalling Technology | 2679 | | 6 |  |
|  | Omnimap anti-rabbit HRP | RTU | 12 | 37 | | Roche Tissue Diagnostics | 760-4311 | |  |  |
|  | Opal 570 | 1:100 | 8 | RT | | Akoya Biosciences | FP1488001KT | |  |  |
| **Cycle 7** | Discovery CC2 | RTU | 8 | 100 | | Roche Tissue Diagnostics | 950-123 | |  | Western Blot and IHC validation from the supplier. Dotted appearance of lysosomes assessed by an in-house pathologist in LUAD tissue. |
|  | Discovery Inhibitor | RTU | N/A | RT | | Roche Tissue Diagnostics | 760-4840 | |  |  |
|  | Beta-galactosidase | 1:150 | 32 | 37 | | Abcam | AB96239 | | *00122836 |  |
|  | Omnimap anti-rabbit HRP | RTU | 16 | 37 | | Roche Tissue Diagnostics | 760-4311 | |  |  |
|  | Opal 540 | 1:200 | 8 | RT | | Akoya Biosciences | FP1494001KT | |  |  |
| **Cycle 8** | Discovery CC2 | RTU | 8 | 100 | | Roche Tissue Diagnostics | 950-123 | |  | Robust validation by the supplier with Western Blot and IHC.  Cytoplasm expression level assessed by an in-house pathologist after in-house validation on LUAD tissue. |
|  | Discovery Inhibitor | RTU | N/A | RT | | Roche Tissue Diagnostics | 760-4840 | |  |  |
|  | Ribosomal Protein S6 (5G10) | 1:50 | 60 | 37 | | Cell Signalling Technology | 2217 | | 13 |  |
|  | Ultramap anti-rabbit HRP | RTU | 12 | 37 | | Roche Tissue Diagnostics | 05269717001 | |  |  |
|  | Opal 480 | 1:50 | 8 | RT | | Akoya Biosciences | FP1500001KT | |  |  |
| **Cycle 9** | Discovery CC2 | RTU | 8 | 100 | | 100 | Roche Tissue Diagnostics | |  | Robust validation from the supplier including IHC DAB in human invasive ductal carcinoma. Recommended parameters used. Positive epithelial cell staining in LUAD tissue assessed by an in-house pathologist. |
|  | Discovery Inhibitor | RTU | N/A | RT | | Roche Tissue Diagnostics | 760-4840 | |  |  |
|  | Pan-cytokeratin (AE1/AE3) | 1:250 | 28 | 37 | | Leica Biosystems | AE1/AE3-601-L-CE | | 6097192 |  |
|  | Omnimap anti-mouse HRP | RTU | 12 | 37 | | Roche Tissue Diagnostics | 760-4310 | |  |  |
|  | TSA-DIG (manual application) | 1:100 | 12 | RT | | Akoya Biosciences | FP1502001KT | |  |  |
|  | Opal 780 (manual application) | 1:10 | 60 | RT | | Akoya Biosciences | FP1501001KT | |  |  |
|  | QD DAPI (three drops) | RTU | 24 | RT | | Roche Tissue Diagnostics | 05268826001 | |  |  |
|  | **Bulk reagents** | | | | | | | | | |
|  | **Reagent** | | | | **Supplier** | | | **Catalogue Number** | | |
|  | Discovery wash | | | | Roche Tissue Diagnostics | | | 07311079001 | | |
|  | Reaction Buffer | | | | Roche Tissue Diagnostics | | | 05353955001 | | |
|  | Liquid Coverslip | | | | Roche Tissue Diagnostics | | | 05264839001 | | |
|  | Antibody Diluent | | | | DAKO | | | K8006 | | |
|  | TSA Diluent | | | | Akoya Biosciences | | | FP1498 | | |
|  | Diamond Prolong Mountant | | | | ThermoFisher Scientific | | | P36970 | | |
|  | TOMO hydrophilic adhesive slides | | | | Matsunami | | | 0808228600 | | |

Key: IHC, immunohistochemistry; DAB, 3,3'-Diaminobenzidine; LUAD, lung adenocarcinoma; KO/KD, knock out/knock down; FFPE, formalin-fixed paraffin embedded; CE-IVD, European *In vitro* diagnostic; RT, room temperature; RTU, ready to use; HRP, horseradish peroxidase; TSA, tyramide signal amplification; DIG, deposition of digoxigenin; Ig, immunoglobin; CC1/2, cell conditioning 1/2.

Panels with ‘*’ depicts the panels used for the deep learning training of PixlMap.
