# Supplementary material for: PixlMap: A generalisable pixel classifier for cellular phenotyping in multiplex immunofluorescence images
Source: PLoS One. 2025 Dec 3;20(12):e0317865. doi: 10.1371/journal.pone.0317865 (PMC12674528; doi:10.1371/journal.pone.0317865)
Supplement: Attachment — Submitted filename: Response to reviewers.pdf [file pone.0317865.s005.pdf]

Rebuttal letter

Journal Requirements:

When submitting your revision, we need you to address these additional requirements.

1.Please ensure that your manuscript meets PLOS ONE's style requirements, including those for file naming. The PLOS ONE style templates can be found at

https://journals.plos.org/plosone/s/file?id=wjVg/PLOSOne_formatting_sample_main_body.pdf and

https://journals.plos.org/plosone/s/file?id=ba62/PLOSOne_formatting_sample_title_authors_affiliations.pdf

This has been completed, thank you.

2. Please note that PLOS ONE has specific guidelines on code sharing for submissions in which author-generated code underpins the findings in the manuscript. In these cases, all author-generated code must be made available without restrictions upon publication of the work. Please review our guidelines at https://journals.plos.org/plosone/s/materials-and-software-sharing#loc-sharing-code and ensure that your code is shared in a way that follows best practice and facilitates reproducibility and reuse.

We will make the code openly available upon acceptance for publication (DOI https://doi.org/10.5281/zenodo.15593393). The Visiopharm APPs used to generate all figures will also be made available upon publication (DOI https://doi.org/10.5281/zenodo.15593785).

3. Please note that funding information should not appear in any section or other areas of your manuscript. We will only publish funding information present in the Funding Statement section of the online submission form. Please remove any funding-related text from the manuscript.

This has been addressed, thank you.

4. We note that the grant information you provided in the ‘Funding Information’ and ‘Financial Disclosure’ sections do not match.

When you resubmit, please ensure that you provide the correct grant numbers for the awards you received for your study in the ‘Funding Information’ section.

Thank you for bringing this to our attention, this has now been corrected.

5. Thank you for stating the following in the Competing Interests section:

“I have read the journal's policy and the authors of this manuscript have the following competing interests:DM is an employee of Visiopharm. All other authors declare no conflict of interest.”

We note that one or more of the authors are employed by a commercial company: Visiopharm

a. Please provide an amended Funding Statement declaring this commercial affiliation, as well as a statement regarding the Role of Funders in your study. If the funding organization did not play a role in the study design, data collection and analysis, decision to publish, or preparation of the manuscript and only provided financial support in the form of authors' salaries and/or research materials, please review your statements relating to the author contributions, and ensure you have specifically and accurately indicated the role(s) that these authors had in your study. You can update author roles in the Author Contributions section of the online submission form.

Please also include the following statement within your amended Funding Statement.

“The funder provided support in the form of salaries for authors [insert relevant initials], but did not have any additional role in the study design, data collection and analysis, decision to publish, or preparation of the manuscript. The specific roles of these authors are articulated in the ‘author contributions’ section.”

If your commercial affiliation did play a role in your study, please state and explain this role within your updated Funding Statement.

b. Please also provide an updated Competing Interests Statement declaring this commercial affiliation along with any other relevant declarations relating to employment, consultancy, patents, products in development, or marketed products, etc.

Within your Competing Interests Statement, please confirm that this commercial affiliation does not alter your adherence to all PLOS ONE policies on sharing data and materials by including the following statement: "This does not alter our adherence to PLOS ONE policies on sharing data and materials.” (as detailed online in our guide for authors http://journals.plos.org/plosone/s/competing-interests) . If this adherence statement is not accurate and there are restrictions on sharing of data and/or materials, please state these. Please note that we cannot proceed with consideration of your article until this information has been declared.

Please include both an updated Funding Statement and Competing Interests Statement in your cover letter. We will change the online submission form on your behalf.

This section has been updated to reflect your comments, thank you for making us aware of this.

6. We note that you have indicated that there are restrictions to data sharing for this study. PLOS only allows data to be available upon request if there are legal or ethical restrictions on sharing data publicly. For more information on unacceptable data access restrictions, please see http://journals.plos.org/plosone/s/data-availability#loc-unacceptable-data-access-restrictions.

Before we proceed with your manuscript, please address the following prompts:

a) If there are ethical or legal restrictions on sharing a de-identified data set, please explain them in detail (e.g., data contain potentially identifying or sensitive patient information, data are owned by a third-party organization, etc.) and who has imposed them (e.g., a Research Ethics Committee or Institutional Review Board, etc.). Please also provide contact information for a data access committee, ethics committee, or other institutional body to which data requests may be sent.

b) If there are no restrictions, please upload the minimal anonymized data set necessary to replicate your study findings to a stable, public repository and provide us with the relevant URLs, DOIs, or accession numbers. For a list of recommended repositories, please see

https://journals.plos.org/plosone/s/recommended-repositories. You also have the option of uploading the data as Supporting Information files, but we would recommend depositing data directly to a data repository if possible.

We will update your Data Availability statement on your behalf to reflect the information you provide.

We have an MTA in place which states that "2.2 The Provider agrees that the Materials are stored in the Biorepository on the strict understanding that, (i) during the Restricted Access Period, the Biorepository will be free to distribute the Materials to the Institutions which have submitted to, and have had approval from, the Biorepository an application to access the Materials and/or Data in accordance with the allocated distribution set out in Schedule 1, and have signed a suitable Material Transfer Agreement with the Biorepository , and (ii) following the Restricted Access Period, the Biorepository will be free to distribute the Materials to Third Party members of the scientific community for the purposes of academic research. The Biorepository agrees to (i) consult on Third Party's requests for the Materials within the local Biorepository Tissue Microarray Management Group of which Dr. John Le Quesne of University of Glasgow, will be a voting member and (ii) only pass Materials onto a Third Party who has signed a Materials Transfer Agreement substantially in the form shown at Schedule 2. In addition, the terms of said Material Transfer Agreement will require that a Third Party wishing to commercially exploit the Materials in any way must first seek a commercial licence from the Provider and any other institution as directed by the Provider in their absolute discretion." In addition, "Third Parties would need to acknowledge the source as LATTICe."

Legally we can therefore provide access to the images used from this tissue collection if 1) We receive an application to request use via the NHS Research Scotland Biorepository portal, 2) We have an MTA for anyone not party to the original agreement to use the images and they would be, 3) expected to include an acknowledgement for the source. We therefore advise that a line be added stating 'For access to the raw data, including images, relating to the LATTICe TMA cohort used in this publication, please contact the Lead Researcher.' To allow us to direct applications for data and image access appropriately.

7. Please include your full ethics statement in the 'Methods' section of your manuscript file. In your statement, please include the full name of the IRB or ethics committee who approved or waived your study, as well as whether or not you obtained informed written or verbal consent. If consent was waived for your study, please include this information in your statement as well.

We have updated this section as requested.

8. Please review your reference list to ensure that it is complete and correct. If you have cited papers that have been retracted, please include the rationale for doing so in the manuscript text, or remove these references and replace them with relevant current references. Any changes to the reference list should be mentioned in the rebuttal letter that accompanies your

revised manuscript. If you need to cite a retracted article, indicate the article's retracted status in the References list and also include a citation and full reference for the retraction notice.

Thank you for raising this point, we have carefully reviewed the reference list and have not discovered any evidence that any references have been retracted.

Additional Editor Comments:

In your revision, please specifically address the following:

- The number of images as well as cells/image that were analyzed for each experiment.

We have updated the text to include this information.

- Details on how mean intensity data were generated, thresholding approaches, expansion methods, and a representative mask from the images for each marker.

We have added these details to the Visiopharm method section.

- Direct comparison of actual cell number results from manual evaluation, PixlMap, and traditional methods for 2-3 markers, along with number of images and approximate number of cells.

We have added the number of cells to the text and figures as requested.

- Address, explain, or fix the issue with field/zoom representation in Figure 4A.

Thank you for pointing this out, this has now been corrected.

- Address in greater detail potential issues with fibroblast detection.

We have expanded on this point in the text; segmenting or phenotyping fibroblasts by any methods is a challenging task due to their variable and elongated shapes and indistinct cell boundaries, and the lack of universal specific antigen markers. Our expectation of success in applying an algorithm trained on 'easier' cell types was limited, but to our surprise the APP performed much better than we expected. We have included this test in the text as it demonstrated the flexibility and performance of the method on a previously unseen cellular morphology which is known to be challenging to interpret.

- Similarly, address potential accuracy issues in identifying CD68+ cells in Figure 5B.

The text has now been updated to address this point. In the example shown we detected low-level but probably physiological CD4 expression within the macrophages, and this signal is likely to have caused the issue highlighted by the reviewer. Having said this, the algorithm still performs well overall.

- Address whether surface markers are more accurately identified versus cytoplasmic markers.

This is an important question, but unfortunately in the current study we do not have the necessary data to test, namely cytoplasmic and surface markers in the same cellular lineage. We will address this in future study.

- Address weakness of metrics in liver tissue comparisons.

We were seeking to test the transferability of PixlMap across species and unseen tissue types, and we feel that the somewhat weaker performance in this out-of-context application is important to report. We clarify this in the text. In addition, we are accumulating evidence, for future publication, that this can be addressed with further species-specific training.

- Clarify language throughout regarding manual versus threshold-based phenotyping.

  The text has been updated to reflect this comment.

Reviewers' comments:

Reviewer's Responses to Questions

Comments to the Author

1. Is the manuscript technically sound, and do the data support the conclusions?

The manuscript must describe a technically sound piece of scientific research with data that supports the conclusions. Experiments must have been conducted rigorously, with appropriate controls, replication, and sample sizes. The conclusions must be drawn appropriately based on the data presented.

Reviewer #1: Partly

Reviewer #2: Partly

Reviewer #3: Yes

---

2. Has the statistical analysis been performed appropriately and rigorously?

Reviewer #1: Yes

Reviewer #2: Yes

Reviewer #3: N/A

---

3. Have the authors made all data underlying the findings in their manuscript fully available?

The PLOS Data policy requires authors to make all data underlying the findings described in their manuscript fully available without restriction, with rare exception (please refer to the Data Availability Statement in the manuscript PDF file). The data should be provided as part of the manuscript or its supporting information, or deposited to a public repository. For example, in addition to summary statistics, the data points behind means, medians and variance measures should be available. If there are restrictions on publicly sharing data—e.g. participant privacy or use of data from a third party—those must be specified.

Reviewer #1: Yes

Reviewer #2: No

Reviewer #3: Yes

---

4. Is the manuscript presented in an intelligible fashion and written in standard English?

PLOS ONE does not copyedit accepted manuscripts, so the language in submitted articles must be clear, correct, and unambiguous. Any typographical or grammatical errors should be corrected at revision, so please note any specific errors here.

Reviewer #1: Yes

Reviewer #2: Yes

Reviewer #3: Yes

---

5. Review Comments to the Author

Please use the space provided to explain your answers to the questions above. You may also include additional comments for the author, including concerns about dual publication, research ethics, or publication ethics. (Please upload your review as an attachment if it exceeds 20,000 characters)

Reviewer #1: While the paper is well presented, it significantly lacks technological novelty. Given the recent advances in generative models, and other advanced mathematical tricks (contrastive learning, different loss functions such as focal loss, ...), I do not entirely subscribe to the idea that you would need significant annotations for this task. In fact, a fairly simple CNN designed with the correct parameters (optimal receptive field) and loss functions could very well be suited here. The article does not talk about the technically novel aspects. I would expect large vision models (such as SAM) to atleast provide a initial ground truth for learning tasks. Unfortunately, I do not think this article is technically solid enough for being published. The methods used (basically a U-Net) are dated, and there's little innovation in terms of architectural design or modelling.

> **We appreciate the time and care taken by Reviewer #1 in reviewing our work, and we regret that our novel aspects were not more clearly expressed, and we have sought to clarify this in the discussion. We acknowledge we are using well established methods that are built into a widely used image analysis platform, and in this sense, there is little methodological novelty. However, we feel that the final algorithm, despite being constructed from familiar elements, is highly novel in its function, and we are pleased to see that such simple and easily adoptable methods produce results which are likely to be of use to the wider community. The novelty of the method comes from the ability to accurately ascribe phenotypes without any**

requirement for whole cell segmentation, in contrast to other currently available methods. This method offers a fast, cost-effective approach to a complex task.

Reviewer #2: The number of images (and cells/image) analyzed should be provided for each experiment.

We would like to thank Reviewer #2 for taking the time to review our work. This raises a good point, and we have altered the text to correct this omission.

Since the performance of the approach is being directly compared to existing approaches (mean intensity), more details are needed on how the mean intensity data were generated (Figure 6H). What thresholding approach was employed? What expansion method was used? Show a representative mask from the images for each marker.

Thank you for highlighting these omissions, we have updated the text accordingly.

Provide actual results in terms of cell numbers of different lineages/markers as determined by manual evaluation, PixlMap, traditional analysis methods. Do this for at least 2 or 3 markers and indicate the number of images and approximate number of cells.

We agree with Reviewer#2 that this will improve clarity of the study, and we have followed their advice.

In Figure 4A the PixlMap probability image does not seem to be the exactly the same field or zoom as the original image. This should be explained or corrected.

Thank you for pointing this out. The figure has now been corrected.

Reviewer #3: Pennie et al. generated a computational tool to classify biomarkers in the multiplexed immunofluorescence images. They note that this tool is inspired by the accuracy of human intuition in identifying positive/negative features. This tool performs classification of biomarkers without the need to define cell boundaries by expanding the nucleus – which is also the primary novelty of this work. The efficacy of this tool was validated in human lung adenocarcinoma samples and murine liver samples.

The manuscript contains sufficient background information for an uninformed reader. Methods are described in sufficient detail and adequate data is shown to draw conclusions. I recommend acceptance of this manuscript. I have the following questions and recommendations.

We appreciate the time and care taken by Reviewer #3 in reviewing our work, in particular we would like to thank them for such detailed feedback.

1. Is fibroblast detection using PixlMap reliable? The authors seem to suggest that it needs further training to be able to detect fibroblasts. I suggest moving Figure 8 to the Supporting Information section if the authors think it needs more work.

Thank you for this suggestion. As discussed in the text, accurate phenotyping of fibroblasts is a very challenging task. We were surprised to find the performance of our method, represented by the heatmaps in figure 8, was much better than expected at dealing with this challenge with no prior training in the same context. We have therefore decided to leave the figure in the main body of the manuscript, and clarify our reasons for doing so.

2. In Figure 5B, it appears that PixlMap fails to identify some CD68+ cells. What is the accuracy and precision in this case?

This is a good point, and we believe it is due to the low level of expression of CD4 in macrophages. We have expanded on the CD68 staining in the discussion to clarify the issue. Overall accuracy and precision are reported in figure 6.

3. In Figure 6, about 32% of true unclassifiable features are predicted as CD4+ by PixlMap? I think it will be important to identify biomarker types that have higher accuracy with PixlMap. For example, authors already mention that spindle shaped cells are not yet accurately identified by PixlMap. Similarly, are surface markers more accurately identified as opposed to cytoplasmic ones?

Fibroblasts are especially challenging because of their variable morphology, indistinct boundaries, and the lack of specific markers- but we were very pleased to see surprisingly good performance of our algorithm which was trained in a very different morphological context. We expand on this in the text.

The issue of surface vs cytoplasmic markers is an important one, but we cannot directly address it at present as we do not have surface and cytoplasmic marker data from cells in the same lineage. We will be sure to examine this in the future.

4. Provide a comparison of the weighted metrics (F1 score, accuracy, precision, and recall) of existing models with your model.

We absolutely appreciate the need for cross-method comparison. However, suitable comparators are not easy to identify; on balance, we feel that threshold-based phenotyping and expert human judgement are the most important and comparable methods, as well as providing the most important 'gold standards'. We did consider including comparison with unsupervised clustering, but the practicalities of making meaningful comparison between cell categories generated in such different ways would make such a comparison hard to perform and interpret.

There are plenty of tools that do some form of phenotyping, but all of them are reliant of cell segmentation. Furthermore, they're all command line tools giving them a certain inaccessibility to non-computationally trained people doing image analysis. We wanted to develop something using commercially available and GUI-friendly software. As such we benchmark against methods that can also be done

within similar confines eg. visual thresholding and the 'real' ground truth ie. pathology annotations.

5. Lines 50 and 51: The authors only talk about tumor microenvironment as a driver to quantify multiple biomarkers simultaneously. There is a need to perform similar analyses in many other non-cancer studies. I suggest changing the sentence to incorporate other applications.

Thank you for raising this important point. This has now been altered in the text.

6. In the murine liver tissue, the accuracy of the model is significantly lower than in human tissue. The F1 scores are also about 0.3 at all probability thresholds. It is understandable that the model is not trained on murine tissues. However, please justify the inclusion of this data with weak metrics in the main manuscript.

As discussed above in our comments to the editor, we were seeking to test the transferability of PixlMap across species and unseen tissue types. We acknowledge that the performance of PixlMap in murine models shown is weaker than the human data set, but we are sufficiently impressed with its out-of-context performance to feel its inclusion warranted.

7. In Figure 6H, the caption mentions "Confusion matrix comparing PixlMap and threshold-based phenotyping using mean pixel intensities of each marker". However, data is shown for Manual annotation vs. threshold-based phenotyping. Please clarify.

Thank you for highlighting this error, this has been corrected in the text.

---

6. PLOS authors have the option to publish the peer review history of their article (what does this mean?). If published, this will include your full peer review and any attached files.

If you choose "no", your identity will remain anonymous but your review may still be made public.

Do you want your identity to be public for this peer review? For information about this choice, including consent withdrawal, please see our Privacy Policy.

Reviewer #1: No

Reviewer #2: No

Reviewer #3: No

[NOTE: If reviewer comments were submitted as an attachment file, they will be attached to this email and accessible via the submission site. Please log into your account, locate the manuscript record, and check for the action link "View Attachments". If this link does not appear, there are no attachment files.]

While revising your submission, please upload your figure files to the Preflight Analysis and Conversion Engine (PACE) digital diagnostic tool, https://pacev2.apexcovantage.com/. PACE helps ensure that figures meet PLOS requirements. To use PACE, you must first register as a user. Registration is free. Then, login and navigate to the UPLOAD tab, where you will find detailed instructions on how to use the tool. If you encounter any issues or have any questions when using PACE, please email PLOS at figures@plos.org. Please note that Supporting Information files do not need this step.
